# Mental Health im Kindesalter: der Einfluss von Mediennutzung, Erziehungsverhalten und elterlichem Stresserleben – eine Sekundärdatenanalyse von KiGGS- und BELLA-Daten

**DOI:** 10.1007/s00103-023-03727-y

**Published:** 2023-06-15

**Authors:** Hannah Lea Jörren, Hannah Schmidt, Anne Kaman, Ulrike Ravens-Sieberer, Hans-Jürgen Rumpf, Silke Pawils

**Affiliations:** 1 Klinik für Psychiatrie und Psychotherapie, Universität zu Lübeck, Ratzeburger Allee 160, 23538 Lübeck, Deutschland; 2 Klinik für Kinder- und Jugendmedizin, Universität zu Lübeck, Lübeck, Deutschland; 3 Zentrum für Psychosoziale Medizin, Klinik für Kinder- und Jugendpsychiatrie, -psychotherapie und -psychosomatik, Universitätsklinikum Hamburg-Eppendorf, Hamburg, Deutschland; 4 Institut für Medizinische Psychologie, Universitätsklinikum Hamburg-Eppendorf, Hamburg, Deutschland

**Keywords:** KiGGS-Studie, Kindheit, Medienkonsum, Psychische Auffälligkeiten, Elterliche Variablen, KiGGS study, Childhood, Media use, Mental health problems, Parental variables

## Abstract

**Hintergrund:**

Studien zeigen einen Zusammenhang zwischen hoher Mediennutzung und psychischen Auffälligkeiten im Kindesalter. Unklar ist jedoch die Rolle von möglichen weiteren Faktoren, die diesen Zusammenhang beeinflussen. Das Ziel der Studie war die Prüfung von Zusammenhängen zwischen psychischen Auffälligkeiten, hoher Mediennutzung, elterlichem Stresserleben sowie inkonsistentem und positivem Erziehungsverhalten.

**Methoden:**

Auf Basis des KiGGS- und BELLA-Datensatzes wurde der Zusammenhang zwischen psychischen Auffälligkeiten und einer hohen Mediennutzung bei Vorschulkindern (Alter: 3–5 Jahre, *n* = 417) und Schulkindern (Alter: 7–13 Jahre, *n* = 239) mittels logistischer Regressionen quer- und längsschnittlich untersucht. Kontrollvariablen waren sozioökonomischer Status, Geschlecht des Kindes und der Eltern, elterliches Stresserleben sowie inkonsistentes und positives Erziehungsverhalten.

**Ergebnisse:**

Im Querschnitt zeigten sich bei Vorschulkindern Zusammenhänge zwischen psychischen Auffälligkeiten mit einer hohen Mediennutzung (OR = 3,02; *p* = 0,003), elterlichem Stresserleben (OR = 17,00; *p* < 0,001) und positivem Erziehungsverhalten (OR = 0,24; *p* < 0,001). Im Längsschnitt zeigte sich bei Schulkindern ein Zusammenhang zwischen psychischen Auffälligkeiten mit elterlichem Stresserleben (OR = 4,04; *p* < 0,001). Sozioökonomischer Status, Geschlecht des Kindes und Geschlecht der Eltern standen nicht im Zusammenhang mit psychischen Auffälligkeiten.

**Diskussion:**

Neben den Wirkmechanismen der digitalen Medien scheinen elterliche Variablen entscheidend für die psychische Gesundheit im Kindesalter zu sein. Sie sollten bei einer ganzheitlichen Betrachtung kindlicher psychischer Gesundheit im Sinne einer Stärkung der elterlichen Kompetenzen Berücksichtigung finden.

**Zusatzmaterial online:**

Zusätzliche Informationen sind in der Online-Version dieses Artikels (10.1007/s00103-023-03727-y) enthalten.

## Abstract (en)

**Background:**

Previous studies established a link between high screen time and mental health problems in childhood. The role of possible influencing factors is currently unclear. This study aims at testing correlations among mental health problems, high screen time, parenting stress, and inconsistent and positive parenting behavior.

**Methods:**

This study is based on data from the KiGGS and BELLA studies. For the present study, data from preschool children (age: 3–5 years, *N* = 417) and school children (age: 7–13 years, *N* = 239) were analyzed. Binary-logistic regressions were carried out to test for correlations between high screen time and children’s mental health problems in cross-sectional and longitudinal analyses. Socioeconomic status, the child’s gender, the parent’s gender, parenting stress, and inconsistent and positive parenting behavior were used as control variables.

**Results:**

In the cross-sectional analysis, mental health problems in preschool children were associated with high screen time (OR = 3.02; *p* = 0.003), parenting stress (OR = 17.00; *p* < 0.001), and positive parenting behavior (OR = 0.24; *p* < 0.001). In the longitudinal analysis, mental health problems in school children were associated with parenting stress (OR = 4.04; *p* < 0.001). Socioeconomic status and the child’s and parent’s gender were at no point associated with mental health problems.

**Discussion:**

The sole existence of high screen time cannot explain mental health problems in children. Parental variables seem to be critical for children’s mental health and should be considered in a holistic apporach on children’s mental health in terms of strengthening parental competencies.

## Hintergrund

In den letzten Jahrzehnten hat sich das Krankheitsspektrum im Kinder- und Jugendbereich von den somatischen zu den psychischen Erkrankungen verschoben. Daher werden Verhaltensprobleme und psychische Erkrankungen in der Pädiatrie als „neue Morbiditäten“ bezeichnet. In Deutschland weisen laut der „Studie zur Gesundheit von Kindern und Jugendlichen in Deutschland (KiGGS)“ 17,2 % der Kinder und Jugendlichen diagnostisch oder klinisch relevante Anzeichen für psychische Auffälligkeiten (PA) auf [1]. Zu diesen PA zählen Depression, Angst und die Aufmerksamkeitsdefizit‑/Hyperaktivitätsstörung (ADHS) mit Prävalenzen von jeweils 11 %, 10 % und 5 % im Elternbericht [2]. Diese Auffälligkeiten können lebenslange Folgen wie soziale Funktionsdefizite und schlechtere akademische Leistungen nach sich ziehen [3].

Als neues Phänomen im Kontext von Verhaltenssuchterkrankungen wird eine hohe Mediennutzung (MN) aktuell kontrovers diskutiert. Ein offiziell anerkanntes Störungsbild besteht in Form der „Internet Gaming Disorder“ [4] bislang nur für das Erwachsenenalter. Im Kindesalter können daher nur Empfehlungen von Mediennutzungszeiten zu der Beurteilung einer möglichen Problematik herangezogen werden. Für Kinder im Vorschulalter wird eine maximale MN von 30 min pro Tag empfohlen [5]. Erhobene Nutzungszeiten überschreiten diese Empfehlung meist: 2‑ bis 5‑jährige Kinder in Deutschland verbringen zwischen 55 min und 86 min täglich mit der Nutzung digitaler Medien [6]. Für Kinder im Schulalter gibt es weniger Konsens über Empfehlungen für die Nutzungsdauer [7]. Die Empfehlung der Europäischen Union (EU) liegt bei maximal 60 min bis 90 min täglich [8]. 6‑ bis 13-Jährige in Deutschland liegen mit einer Nutzung digitaler Medien von 159 min pro Tag [9] über dieser Empfehlung. Während der COVID-19-Pandemie erhöhten sich die Nutzungszeiten digitaler Medien bei Kindern [10]. Da frühere Studien einen positiven Zusammenhang zwischen einer hohen MN und emotionalen Symptomen sowie Verhaltensstörungen [11] feststellen konnten, sollte diese Entwicklung aufmerksam beobachtet werden.

Der Zugang zu digitalen Medien ist in Deutschland bereits im Vorschulalter gegeben: 19 % besitzen einen Computer oder haben eigenständigen Zugang zu diesem [6]. Hier gibt es jedoch Unterschiede, abhängig vom sozioökonomischen Status (SES): In Familien mit einem niedrigen SES besitzen mehr Kinder ein technisches Endgerät [12]. Ein niedriger SES stellt ebenfalls einen Risikofaktor für psychische Auffälligkeiten dar [2]. Neben dem SES gibt es Hinweise auf einen Einfluss des Geschlechts des Kindes sowohl auf die MN als auch auf PA: Jungen weisen eine leicht höhere Bildschirmzeit auf [13] und sind häufiger von PA betroffen [1]. Ob das kindliche Geschlecht ebenfalls einen Einfluss auf den Zusammenhang zwischen PA und einer hohen MN nimmt, ist bislang nicht eindeutig geklärt [14, 15]. Da es Hinweise auf Unterschiede in der Beurteilung der PA von Kindern durch Mütter und Väter gibt [16], wurde das Geschlecht des interviewten Elternteils in der vorliegenden Arbeit ebenfalls als Kontrollvariable mit aufgenommen.

Weitere Einflussfaktoren für PA in der kindlichen Umwelt sind unter anderem elterliches Stresserleben sowie elterliches Erziehungsverhalten. Elterliches Stresserleben scheint im Zusammenhang zu stehen mit kindlichen PA [17]. Aufgrund der Stabilität von PA [17] scheint eine Betrachtung zu unterschiedlichen Zeitpunkten in der Kindheit angemessen. Bisherige Studien weisen auf einen Zusammenhang zwischen elterlichem Verhalten und PA [18] hin. Inkonsistentes Erziehungsverhalten^1^ steht im Zusammenhang mit Verhaltensproblemen [18, 19]; ein positives Erziehungsverhalten^2^ ist assoziiert mit einer besseren Emotionsregulation und weniger Verhaltensproblemen [20].

Es bleibt unklar, wie PA mit einer hohen MN in unterschiedlichen Altersgruppen zusammenhängen und ob ein vorhandener Zusammenhang unter Hinzunahme von SES, Geschlecht des Kindes, Geschlecht des interviewten Elternteils, elterlichem Stresserleben sowie positivem und inkonsistentem Erziehungsverhalten Bestand hat. Dies führt zu den folgenden Hypothesen:

1. *Vorschulalter (Querschnitt)*
  - PA im Alter von 3–5 Jahren sind assoziiert mit einer hohen MN.
  - Dieser Zusammenhang besteht unter Hinzunahme der Kontrollvariablen SES, Geschlecht des Kindes, Geschlecht des interviewten Elternteils, elterliches Stresserleben, positives und inkonsistentes Erziehungsverhalten.
2. *Schulalter (Längsschnitt)*
  - PA bei Kindern im Alter von 11–13 Jahren zum Follow-up sind assoziiert mit einer hohen MN zur Baseline im Alter von 7–10 Jahren.
  - Dieser Zusammenhang besteht unter Hinzunahme der Kontrollvariablen SES, Geschlecht des Kindes, Geschlecht des interviewten Elternteils, elterliches Stresserleben, positives und inkonsistentes Erziehungsverhalten.

## Methoden

### Teilnehmende und Vorgehen

**Figure 1:**
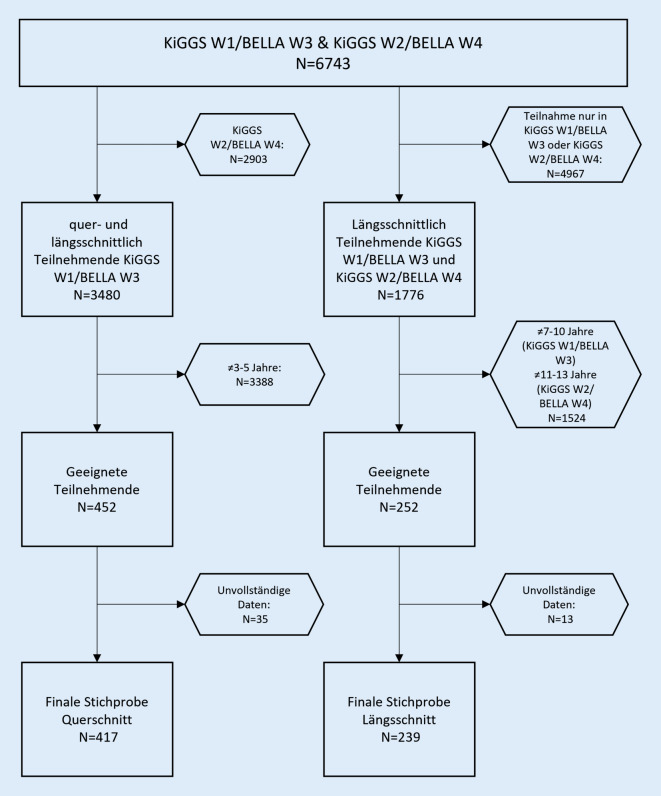

Die Datengrundlage der vorliegenden Studie entstammt der repräsentativen „Studie zur Gesundheit von Kindern und Jugendlichen in Deutschland (KiGGS)“ [21, 22] sowie dem Modul „Befragung zum seelischen Wohlbefinden und Verhalten (BELLA)“ [23, 24]. Verwendet wurden Daten aus KiGGS Welle 1 (erhoben von 2009 bis 2012) und KiGGS Welle 2 (erhoben von 2014 bis 2017) sowie Daten aus den BELLA Wellen 3 (2009–2012) und 4 (2014–2017), die parallel erhoben wurden (alle Teilnehmenden der BELLA-Studie nahmen auch an KiGGS teil). Es wurden 2 Stichproben für Quer- und Längsschnittanalysen gezogen. Für die Querschnittsanalysen im Vorschulalter kamen Teilnehmende aus KiGGS Welle 1/BELLA Welle 3 im Alter von 3 bis 5 Jahren mit vollständigen Daten für den SES, den Strengths and Difficulties Questionnaire (SDQ), die Medienitems, die Skalen positives und inkonsistentes Erziehungsverhalten des Alabama Parenting Questionnaire für Kinder im Grundschulalter (DEAPQ-EL-GS) und die Skala für elterliches Stresserleben des Elternstressfragebogens (ESF) infrage (insgesamt *n* = 417). Für die Längsschnittanalysen im Schulalter kamen Teilnehmende infrage, die an KiGGS Welle 1/BELLA Welle 3 (Baseline) im Alter von 7 bis 10 Jahren und an KiGGS Welle 2/BELLA Welle 4 (Follow-up) im Alter von 11 bis 13 Jahren teilgenommen haben und vollständige Daten für den SES und SDQ zur Baseline und zum Follow-up sowie vollständige Daten für die Medienitems, die Skalen positives und inkonsistentes Erziehungsverhalten des DEAPQ-EL-GS und die Skala elterliches Stresserleben des ESF zur Baseline aufweisen (insgesamt *n* = 239; Abb. 1). Die Daten der KiGGS-Studie wurden für die Instrumente SDQ, Medienitems und Erfassung des SES genutzt. Für die Instrumente DEAPQ-EL-GS und ESF wurden Daten des BELLA-Moduls verwendet. Alle verwendeten Instrumente und Skalen wurden im Elternbericht erhoben.

### Instrumente

#### PA: Strengths and Difficulties Questionnaire (SDQ, KiGGS).

Der SDQ misst PA auf 5 Skalen mit jeweils 5 Items: Hyperaktivität, emotionale Symptome, Verhaltensprobleme, Probleme mit Gleichaltrigen und prosoziales Verhalten. Ein allgemeiner Schwierigkeitswert von 0 bis 40 kann durch Aufsummieren der Werte für Hyperaktivität, emotionale Symptome, Verhaltensprobleme und Probleme mit Gleichaltrigen ermittelt werden [25]. In der vorliegenden Studie wurde der binäre Wert („normal“ vs. „grenzwertig/auffällig“ mit einem Cut-off von 13) verwendet. Die interne Konsistenz für die Gesamtskala der Elternversion ist hoch (α = 0,81; [26]).

#### MN: Medienitems (KiGGS).

Die MN wurde mit Items gemessen, die vom KiGGS-Studienteam entwickelt wurden:

Wie lange beschäftigt sich Ihr Kind durchschnittlich pro Tag mit folgenden Dingen?

1. Fernsehen und Video
2. Spielekonsole
3. Computer/Internet

Die Antwortkategorien für jede Skala sind 1 = überhaupt nicht, 2 = bis zu 1 h pro Tag, 3 = bis zu 2 h pro Tag, 4 = bis zu 3 h pro Tag, 5 = bis zu 4 h pro Tag, 6 = mehr als 4 h pro Tag. Die Skalen wurden mit den Werten 0; 0,5; 1,5; 2,5; 3,5; 5 transformiert, um eine metrische Variable zu erstellen [27]. Die Skalen wurden zu einem Gesamtwert für die MN in Stunden pro Tag addiert. Eine hohe MN bei Vorschulkindern wurde anhand der Leitlinien der Bundeszentrale für gesundheitliche Aufklärung (BZgA) definiert: Die BZgA empfiehlt eine maximale tägliche MN von 30 min für 3‑ bis 5‑Jährige [5]. Eine hohe MN bei Schulkindern wurde für 2 Altersgruppen definiert: 7‑ bis 10-Jährige sollten nach einer EU-Empfehlung digitale Medien maximal 60 min täglich nutzen [28]. Für 12- bis 13-Jährige wurde die durchschnittliche MN aus der KIM-Studie (Kindheit, Internet, Medien) 2016 herangezogen: Der deutsche Mittelwert für die Fernseh‑, Videospiel‑, Computer- und Internetnutzung der 12- bis 13-Jährigen liegt bei 214 min täglich [9].

#### Soziodemografie (KiGGS).

Die Soziodemografie wurde mittels der Angaben zu Geschlecht des Kindes und Geschlecht des interviewten Elternteils sowie anhand des überarbeiteten SES-Index der Eltern erfasst. Die verwendeten Variablen (SES, PA, MN, elterliches Stresserleben, positives und inkonsistentes Erziehungsverhalten) wurden auf mögliche kindliche Geschlechtsunterschiede hin untersucht. Der überarbeitete SES-Index ist ein multidimensionaler Indexwert [29]. Er ist die Summe aus 3 metrischen Komponenten: Schulbildung und berufliche Qualifikation, berufliche Stellung und Netto-Äquivalenzeinkommen. Für die Analyse des SES wird der Gesamtindexwert in Quintile unterteilt. Das erste Quintil steht für einen niedrigen SES, das zweite bis vierte Quintil für einen mittleren SES und das fünfte Quintil für einen hohen SES [29].

#### Elterliches Stresserleben: Elternstressfragebogen (ESF, BELLA).

Der ESF erfasst elterliches Stresserleben und ist sowohl für Eltern von Vorschulkindern als auch von Kindern von der 1. bis zur 6. Klasse geeignet [30]. Der Fragebogen besteht aus 4 Skalen mit insgesamt 38 Items: elterliches Stresserleben, Rollenrestriktion, soziale Unterstützung und Partnerschaft [31]. Für diese Studie wurde die Skala „elterliches Stresserleben“ verwendet. Diese besteht aus 17 Items mit einer 4‑stufigen Skala (0 = trifft nicht zu, 1 = trifft kaum zu, 2 = trifft etwas zu, 3 = trifft genau zu). Im vorliegenden Modell wurde der Mittelwert der Rohwerte verwendet. Dieser kann zwischen 0 und maximal 3 liegen. Die interne Konsistenz der Skalen ist mit α = 0,76–0,92 als hoch einzustufen [31].

#### Erziehungsverhalten: Deutsche erweiterte Version des Alabama Parenting Questionnaire für Kinder im Grundschulalter (DEAPQ-EL-GS, BELLA).

Der DEAPQ-EL-GS ist die deutsche Version des Alabama Parenting Questionnaire, modifiziert für Eltern von Grundschulkindern [19]. Er erfasst das Erziehungsverhalten aus der Sicht der Eltern. Der DEAPQ-EL-GS enthält 7 Skalen mit insgesamt 40 Items [19]. In dieser Studie wurden 2 Skalen verwendet: „positives Erziehungsverhalten“ und „inkonsistentes Erziehungsverhalten“, bestehend aus jeweils 6 Items mit einer 5‑stufigen Skala (1 = fast nie, 5 = fast immer). In der vorliegenden Studie wurde der Mittelwert der Rohwerte verwendet. Dieser kann Werte zwischen 1 und maximal 5 annehmen. Die interne Konsistenz der verwendeten Skalen ist mit α = 0,84 für das positive Erziehungsverhalten und α = 0,72 für das inkonsistente Erziehungsverhalten als akzeptabel einzustufen [19].

#### Statistische Analysen.

Die statistischen Analysen wurden mit IBM SPSS Statistics 28 (IBM Corp., Armonk, NY, USA) durchgeführt. Neben deskriptiven Analysen wurden, unter Berücksichtigung von SES, Geschlecht des Kindes und des interviewten Elternteils, elterlichem Stresserleben sowie positivem und inkonsistentem Erziehungsverhalten, binär-logistische Regressionen durchgeführt, um einen möglichen Zusammenhang zwischen PA und hoher MN bei Kindern im Vorschulalter und Schulalter zu ermitteln. Alle Hypothesen wurden mit einem Signifikanzniveau von α = 5 % getestet. PA wurden als abhängige Variable untersucht.

## Ergebnisse

### Zusammenhänge im Vorschulalter (Querschnittsanalysen)

Eine Stichprobenbeschreibung sowie deskriptive Daten zu PA, zur MN, zum elterlichen Stresserleben und zum Erziehungsverhalten sind in Tab. 1 zusammengefasst. Eine Korrelationsmatrix findet sich im Onlinematerial 1. Das durchschnittliche Alter in der Vorschulstichprobe lag bei 3,8 Jahren (SD = 0,82) mit einem ausgewogenen Geschlechterverhältnis von 48,9 % Mädchen und 51,1 % Jungen. Der Anteil der Mütter lag mit 88,7 % deutlich über dem der Väter (11,0 %). Das mittlere Alter der Mütter betrug 35,20 Jahre (SD = 5,01), das der Väter 40,42 Jahre (SD = 5,72). Mit je 57,3 % und 36,7 % waren ein mittlerer und hoher SES überproportional vertreten. Der Großteil der Stichprobe zeigte sich hinsichtlich PA unauffällig (91,6 %). Knapp ein Drittel der Vorschulkinder lag über den Mediennutzungsempfehlungen der BZgA von maximal 30 min täglich. Es ließen sich keine kindlichen Geschlechtsunterschiede hinsichtlich der untersuchten Variablen feststellen.

**Table 1:** 

	Gesamt	Weiblich	Männlich	*p*^a^
	(*n* = 417)	(*n* = 204)	(*n* = 213)	
*Alter in Jahren, M (SD)*	3,84 (0,82)	3,81 (0,83)	3,86 (0,82)	0,446
*Sozioökonomischer Status*				0,945
Niedrig, *n (%)*	25 (6,0)	13 (6,4)	12 (5,6)	
Mittel, *n (%)*	239 (57,3)	117 (57,4)	122 (57,3)	
Hoch, *n (%)*	153 (36,7)	74 (36,3)	79 (37,1)	
*Psychische Auffälligkeiten*				0,881
Unauffällig, *n (%)*	382 (91,6)	187 (91,7)	195 (91,5)	
Grenzwertig, *n (%)*	17 (4,1)	9 (4,4)	8 (3,8)	
Auffällig, *n (%)*	18 (4,3)	8 (3,9)	10 (4,7)	
*Mediennutzung*				0,248
Unauffällig, *n (%)*	281 (67,4)	143 (70,1)	138 (64,8)	
Auffällig, *n (%)*	136 (32,6)	61 (29,9)	75 (35,2)	
*Elterliches Stresserleben, M (SD)*	0,86 (0,51)	0,86 (0,50)	0,86 (0,52)	0,929
*Positives Erziehungsverhalten, M (SD)*	4,40 (0,42)	4,43 (0,43)	4,38 (0,42)	0,171
*Inkonsistentes Erziehungsverhalten, M (SD)*	2,29 (0,66)	2,29 (0,66)	2,29 (0,67)	0,814

^a^Chi-Quadrat- und Man-Whitney-U-Test, asymptotische Signifikanz, zweiseitig

Die Ergebnisse der binär-logistischen Regressionen zur Vorhersage von PA bei Vorschulkindern sind in Tab. 2 dargestellt. Eine hohe MN hing, unter Kontrolle von SES und Geschlecht des Kindes und der Eltern, signifikant mit der Auftretenswahrscheinlichkeit für PA zusammen (Modell 1: OR = 3,02; *p* = 0,003). Die Varianzaufklärung durch das Modell war niedrig (Nagelkerkes R^2^ = 0,10), der Zusammenhang zwischen PA und MN demnach eher schwach. Unter der Hinzunahme der Kontrollvariablen elterliches Stresserleben, positives und inkonsistentes Erziehungsverhalten blieb der Zusammenhang zwischen einer hohen MN und PA bestehen (Modell 2: OR = 3,06; *p* = 0,011; Modell 3: OR = 2,92; *p* = 0,005; Modell 4: OR = 2,70; *p* = 0,008). Positives Erziehungsverhalten (OR = 0,24; *p* < 0,001) und elterliches Stresserleben (OR = 17,00; *p* < 0,001) zeigten einen Zusammenhang mit PA. Modell 2 unter Hinzunahme des elterlichen Stresserlebens weist die größte Effektstärke auf (Nagelkerkes R^2^ = 0,40). Die Kontrollvariablen SES und Geschlecht des Kindes und der Eltern wurden in keinem Modell signifikant.

**Table 2:** 

	OR^a^	*p*-Wert	95 % KI für OR	Varianzaufklärung^b^	Omnibustest
*Modell 1*				0,10	X^2^ = 17,67, df = 4, *p* = 0,001
Hohe Mediennutzung	3,02	0,003*	1,47; 6,24		
Sozioökonomischer Status	0,91	0,102	0,82; 1,02		
Geschlecht des Kindes	0,97	0,942	0,48; 1,98		
Geschlecht der Eltern	5,64	0,095	0,74; 43,03		
*Modell 2*				0,40	X^2^ = 80,13, df = 5, *p* < 0,001
Hohe Mediennutzung	3,06	0,011*	1,29; 7,28		
Elterliches Stresserleben	17,00	< 0,001*	7,37; 39,24		
Sozioökonomischer Status	0,93	0,286	0,82; 1,06		
Geschlecht des Kindes	1,13	0,771	0,49; 2,61		
Geschlecht der Eltern	2,62	0,366	0,33; 21,07		
*Modell 3*				0,16	X^2^ = 29,93, df = 5, *p* < 0,001
Hohe Mediennutzung	2,92	0,005*	1,38; 6,17		
Positives Erziehungsverhalten	0,24	< 0,001*	0,10; 0,54		
Sozioökonomischer Status	0,92	0,123	0,83; 1,03		
Geschlecht des Kindes	1,03	0,939	0,50; 2,14		
Geschlecht der Eltern	6,34	0,079	0,81; 49,67		
*Modell 4*				0,11	X^2^ = 20,97, df = 5, *p* < 0,001
Hohe Mediennutzung	2,70	0,008*	1,29; 5,66		
Inkonsistentes Erziehungsverhalten	1,64	0,069	0,96; 2,79		
Sozioökonomischer Status	0,92	0,140	0,82; 1,03		
Geschlecht des Kindes	0,97	0,936	0,48; 1,99		
Geschlecht der Eltern	6,36	0,076	0,82; 49,01		

* zeigt signifikantes Ergebnis an

^a^Odds Ratio

^b^Nagelkerkes R

### Prädiktoren im Schulalter (Längsschnittanalysen)

Eine Stichprobenbeschreibung sowie deskriptive Daten zum elterlichen Stresserleben und zum Erziehungsverhalten zur Baseline sowie zu PA und zur MN zu Baseline und Follow-up sind in Tab. 3 zusammengefasst. Eine Korrelationsmatrix findet sich im Onlinematerial 2. Das Alter der Schulkinder betrug durchschnittlich 7,6 Jahre (SD = 0,60) zur Baseline und 12,5 Jahre (SD = 0,60) zum Follow-up. Mit 49,8 % Mädchen und 50,2 % Jungen war das Geschlechterverhältnis ausgeglichen. Der Anteil der Mütter lag mit 90,8 % deutlich über dem der Väter (8,8 %). Das mittlere Alter der Mütter betrug 38,94 Jahre (SD = 4,73), das der Väter 45,05 Jahre (SD = 6,74). Mittlerer SES (59,4 %) und hoher SES (36,8 %) waren überproportional vertreten.

**Table 3:** 

	Gesamt		Weiblich		Männlich		*P*^a^	
	T0	T1	T0	T1	T0	T1	T0	T1
*Alter in Jahren, M (SD)*	7,62 (0,60)	12,47 (0,60)	7,61 (0,59)	12,45 (0,59)	7,63 (0,61)	12,49 (0,61)	0,850	0,550
*Sozioökonomischer Status*							0,586	0,532
Niedrig, *n (%)*	9 (3,8)	18 (7,5)	6 (5,0)	11 (9,2)	3 (2,5)	7 (5,8)	–	–
Mittel, *n (%)*	142 (59,4)	155 (64,9)	70 (58,8)	74 (62,2)	72 (60,0)	81 (67,5)	–	–
Hoch, *n (%)*	88 (36,8)	66 (27,6)	43 (36,1)	34 (28,6)	45 (37,5)	32 (26,7)	–	–
*Psychische Auffälligkeiten*							0,104	0,170
Unauffällig, *n (%)*	208 (87,0)	214 (89,5)	109 (91,6)	111 (93,3)	99 (82,5)	103 (85,8)	–	–
Grenzwertig, *n (%)*	17 (7,1)	16 (6,7)	6 (5,0)	5 (4,2)	11 (9,2)	11 (9,2)	–	–
Auffällig, *n (%)*	14 (5,9)	9 (3,8)	4 (3,4)	3 (2,5)	10 (8,3)	6 (5,0)	–	–
*Mediennutzung*							0,027*	0,061
Unauffällig, *n (%)*	84 (35,1)	120 (50,2)	50 (42,0)	67 (56,3)	34 (28,3)	53 (44,2)	–	–
Auffällig, *n (%)*	155 (64,9)	119 (49,8)	69 (58,0)	52 (43,7)	86 (71,7)	67 (55,8)	–	–
*Elterliches Stresserleben, M (SD)*	0,77 (0,57)	k. A.	0,68 (0,52)	k. A.	0,86 (0,64)	k. A.	0,041*	k. A.
*Positives Erziehungsverhalten, M (SD)*	4,19 (0,50)	k. A.	4,19 (0,48)	k. A.	4,19 (0,53)	k. A.	0,672	k. A.
*Inkonsistentes Erziehungsverhalten, M (SD)*	2,22 (0,64)	k. A.	2,15 (0,66)	k. A.	2,29 (0,62)	k. A.	0,126	k. A.

*k.* *A.* keine Angabe. * zeigt signifikantes Ergebnis an

^a^Chi-Quadrat- und Man-Whitney-U-Test, asymptotische Signifikanz, zweiseitig

Hinsichtlich PA zeigte sich der Großteil der Stichprobe sowohl zur Baseline (T0) als auch zum Follow-up (T1) unauffällig (T0: 87,0 %; T1: 89,5 %). Knapp 2 Drittel der Schulkinder lagen zur Baseline über der Nutzungsempfehlung der EU von maximal 60 min digitale Medien täglich. Zum Follow-up lagen 49,8 % der Teilnehmenden über dem deutschen Durchschnitt der MN. Signifikante kindliche Geschlechtsunterschiede wurden bei der MN zur Baseline (*p* = 0,027) und dem elterlichen Stresserleben zur Baseline (*p* = 0,041) festgestellt, wobei Jungen bzw. deren Eltern stärker betroffen waren.

Die Ergebnisse der binär-logistischen Regressionen zur Vorhersage von PA bei Kindern im Schulalter sind in Tab. 4 dargestellt. Nach Kontrolle von PA zur Baseline sowie von SES und Geschlecht des Kindes und der Eltern zeigte sich kein Zusammenhang zwischen einer hohen MN zur Baseline und PA zum Follow-up (OR = 1,13; *p* = 0,806). Die Kontrollvariable elterliches Stresserleben (OR = 4,04; *p* < 0,001) erwies sich als signifikanter Prädiktor von PA zum Follow-up. Positives Erziehungsverhalten (OR = 0,89; *p* = 0,786) und inkonsistentes Erziehungsverhalten (OR = 1,70; *p* = 0,147) zur Baseline zeigten keine signifikanten Zusammenhänge mit PA zum Follow-up. Die Kontrollvariablen SES und Geschlecht des Kindes und der Eltern waren in keinem Modell signifikante Prädiktoren für PA.

**Table 4:** 

Prädiktoren psychischer Auffälligkeiten zu T1	OR^a^	*p*-Wert	95 % KI für OR	Varianzaufklärung^b^	Omnibustest
*Modell 1*				0,16	X^2^ = 19,15, df = 5, *p* = 0,002
Psychische Auffälligkeiten^c^	7,07	< 0,001*	2,70; 18,49		
Hohe Mediennutzung^c^	1,13	0,806	0,42; 3,02		
Sozioökonomischer Status^c^	1,02	0,757	0,90; 1,17		
Geschlecht des Kindes^c^	0,54	0,198	0,21; 1,38		
Geschlecht der Eltern^c^	1,10	0,901	0,23; 5,43		
*Modell 2*				0,26	X^2^ = 32,14, df = 6, *p* < 0,001
Psychische Auffälligkeiten^c^	2,73	0,079	0,89; 8,34		
Hohe Mediennutzung^c^	1,35	0,578	0,47; 3,84		
Elterliches Stresserleben^c^	4,04	< 0,001*	1,84; 8,90		
Sozioökonomischer Status^c^	1,00	0,932	0,88; 1,16		
Geschlecht des Kindes^c^	0,68	0,439	0,25; 1,82		
Geschlecht der Eltern^c^	1,28	0,775	0,24; 6,89		
*Modell 3*				0,16	X^2^ = 19,22, df = 6, *p* = 0,004
Psychische Auffälligkeiten^c^	6,86	< 0,001*	2,56; 18,37		
Hohe Mediennutzung^c^	1,13	0,802	0,42; 3,03		
Positives Erziehungsverhalten^c^	0,89	0,786	0,38; 2,08		
Sozioökomischer Status^c^	1,02	0,746	0,90; 1,17		
Geschlecht des Kindes^c^	0,55	0,201	0,22; 1,38		
Geschlecht der Eltern^c^	1,11	0,903	0,23; 5,43		
*Modell 4*				0,18	X^2^ = 21,32, df = 5, *p* = 0,002
Psychische Auffälligkeiten^c^	5,40	< 0,001*	1,93; 15,09		
Hohe Mediennutzung^c^	1,04	0,933	0,38; 2,83		
Inkonsistentes Erziehungsverhalten^c^	1,70	0,147	0,83; 3,48		
Sozioökonomischer Status^c^	1,03	0,665	0,90; 1,18		
Geschlecht des Kindes^c^	0,54	0,196	0,21; 1,38		
Geschlecht der Eltern^c^	1,24	0,795	0,24; 6,35		

* zeigt signifikantes Ergebnis an

^a^Odds Ratio

^b^Nagelkerkes R^2^

^c^Zu T0

## Diskussion

Ziel der Studie war es, mögliche Zusammenhänge zwischen einer hohen MN und PA in unterschiedlichen Altersgruppen aufzudecken und unter Hinzunahme soziodemografischer und elterlicher Variablen weiter zu überprüfen. Im Querschnitt zeigten sich Zusammenhänge zwischen PA und einer hohen MN, elterlichem Stresserleben und positivem Erziehungsverhalten bei Vorschulkindern. In der längsschnittlichen Betrachtung zeigte sich kein Zusammenhang zwischen einer hohen MN und PA. Elterliches Stresserleben zur Baseline zeigte einen signifikanten Zusammenhang mit späteren PA.

### Querschnittliche Zusammenhänge zwischen PA, hoher MN und elterlichen Variablen bei Vorschulkindern

In der vorliegenden Studie wurde ein Zusammenhang zwischen PA und einer hohen MN bei 3‑ bis 5‑jährigen Kindern festgestellt. Dieses Ergebnis deckt sich mit früheren Studien in diesem Bereich, wonach eine hohe MN positiv mit emotionalen Symptomen, Hyperaktivität und Verhaltensproblemen korreliert [11]. Ein möglicher Erklärungsansatz für den Zusammenhang lässt sich in der Verdrängung wichtiger Aktivitäten wie sozialer Interaktion [32] durch eine hohe MN finden. In der Interaktion mit Gleichaltrigen und erwachsenen Bezugspersonen erlernen Kinder Selbstregulation, Empathie und soziale Fähigkeiten [33]. Soziale Fähigkeiten hängen negativ mit Verhaltensproblemen zusammen [34]. Eine hohe MN könnte die Gelegenheiten reduzieren, zu denen Kinder wichtige Erfahrungen sammeln und Fähigkeiten erlernen können. Eine weitere Erklärung für den Zusammenhang könnte sein, dass Eltern von Kindern mit externalisierendem und antisozialem Verhalten eine höhere MN autorisieren, um diese zu beruhigen und zu beschäftigen [33]. Der Zusammenhang zwischen PA und einer hohen MN im Vorschulalter spricht dafür, Kindern bereits in diesem Alter einen bewussten Umgang mit Medien zu vermitteln. Eine frühe digitale Medienbildung ist in deutschen Kindertagesstätten jedoch meist unterentwickelt und bedarf einer Verbesserung unter anderem in der technischen Infrastruktur sowie der Ausbildung des pädagogischen Personals [35].

Darüber hinaus hat sich in der vorliegenden Studie gezeigt, dass PA mit elterlichem Stresserleben korrelieren. Dies deckt sich mit früheren Studien [18]. Verhaltensprobleme von Kindern gelten als wichtigster Prädiktor für elterliches Stresserleben [36]. Neben Hinweisen auf einen direkten Zusammenhang zwischen PA und elterlichem Stresserleben [37] gibt es Evidenz für mögliche moderierende Faktoren des Zusammenhangs. Elterliches Stresserleben und kindliche Verhaltensprobleme hängen bei unsicherer, jedoch nicht bei sicherer Eltern-Kind-Bindung zusammen [38]. Kindliche Variablen wie eine hohe Emotionalität können ebenfalls zu einem stärkeren Zusammenhang zwischen elterlichem Stresserleben und PA führen [39].

In der vorliegenden Studie wurde ein signifikanter Zusammenhang zwischen PA und positivem Erziehungsverhalten festgestellt. Die bisherige Evidenz für diesen Zusammenhang ist inkonsistent. Neben Null-Befunden für den Zusammenhang zwischen positivem Erziehungsverhalten und PA bei Kindern [18] lassen sich Zusammenhänge zwischen kindlichen PA auf der einen Seite und elterlicher Wärme sowie autoritativem Erziehungsverhalten auf der anderen Seite finden. Hierbei stehen die kindlichen und elterlichen Variablen in einem wechselseitigen Zusammenhang [40]. Ein möglicher Wirkmechanismus des Zusammenhangs könnte in einer bewussten kindlichen Emotionsregulation zu finden sein [20]. Diese steht in einem positiven Zusammenhang mit positivem elterlichen Erziehungsverhalten und in einem negativen Zusammenhang mit kindlichen Verhaltensproblemen [20]. Auf diese Weise könnte die kindliche Emotionsregulation dem gefundenen Zusammenhang zwischen kindlichen PA und positivem elterlichen Erziehungsverhalten zugrunde liegen.

Auffallend in den Ergebnissen war der fehlende Zusammenhang zwischen PA und dem SES. Frühere Studien konnten zeigen, dass Kinder in Deutschland aus Familien mit einem niedrigen SES mehr als doppelt so häufig von PA betroffen sind wie Kinder aus Familien mit einem hohen SES [1]. Eine mögliche Ursache für das Fehlen des Zusammenhangs könnte in der Unterrepräsentation von Kindern aus Familien mit einem niedrigen SES in der vorliegenden Stichprobe zu finden sein.

Ebenfalls auffällig war der fehlende Zusammenhang zwischen PA und dem Geschlecht des Kindes. Das Geschlecht des Kindes könnte den Zusammenhang zwischen einer hohen MN und PA moderieren. Die Befunde bezüglich des kindlichen Geschlechts als Moderatorvariable sind inkonsistent [41, 42]. Daher werden weitere Studien empfohlen, die das kindliche Geschlecht als mögliche Moderatorvariable untersuchen.

### Längsschnittliche Zusammenhänge zwischen PA, hoher MN und elterlichen Variablen bei Schulkindern

Eine hohe MN zur Baseline war kein Prädiktor für PA im Follow-up. Frühere Studien in diesem Bereich zeigen inkonsistente Ergebnisse: Zum einen zeigen sich nur geringe Zusammenhänge zwischen einer hohen MN in der Kindheit und PA im frühen Jugendalter [43]. Zum anderen zeigt eine andere Untersuchung, dass psychosoziale Probleme bei Kindern, die zu Beginn der Studie mehr Zeit am Bildschirm verbrachten, im Laufe der Zeit stärker zunehmen [41]. In der vorliegenden Studie wurden die Nutzungszeiten digitaler Medien untersucht. Es gibt Hinweise, dass der Zusammenhang zwischen PA und hoher MN von Faktoren wie der Art der Nutzung [42] und den Inhalten der Medien [44] beeinflusst wird. Somit könnte die Form der Erfassung der MN einen Einfluss auf das Feststellen eines Zusammenhangs zwischen einer hohen MN und PA haben.

Die logistischen Regressionsanalysen identifizierten elterliches Stresserleben als Prädiktor für PA. Dieser Befund deckt sich mit früheren Studien [45]. Zwischen Verhaltensproblemen und elterlichem Stresserleben in der frühen und mittleren Kindheit besteht eine transaktionale Beziehung, wobei das elterliche Stresserleben sowohl Vorläufer als auch Folge der Verhaltensprobleme des Kindes sein kann [46]. Das elterliche Stresserleben scheint mit der Selbstwirksamkeit der Eltern zusammenzuhängen: Je höher die Selbstwirksamkeit, desto geringer das elterliche Stresserleben [47]. Deshalb könnten Programme zur Förderung der elterlichen Selbstwirksamkeit eine mögliche Intervention sein, um das elterliche Stresserleben zu reduzieren und damit die Wahrscheinlichkeit von PA bei Kindern teilweise zu verringern.

### Stärken und Limitationen

Trotz der Kombination von Quer- und Längsschnittanalysen weist diese Studie einige Limitationen auf. Angesichts der schnellen Entwicklung der Medien und der damit einhergehenden Veränderung der MN könnten die Daten als vergleichsweise alt kritisiert werden. Daher werden, insbesondere im Hinblick auf die aktuelle Entwicklung durch die COVID-19-Pandemie, weitere Analysen mit neueren Daten empfohlen. So zeigt sich sowohl im Vorschulalter als auch im Schulalter ein Anstieg der Mediennutzungsdauer im Vergleich zu den Jahren vor der Pandemie [10, 48].

Da in der vorliegenden Studie nur die Dauer der MN gemessen wurde, können keine Aussagen über die Auswirkungen von Art der Nutzung und Inhalte der Medien getroffen werden. Beides kann sich jedoch auf den Zusammenhang zwischen MN und PA auswirken [42, 44] und sollte in folgenden Studien Berücksichtigung finden.

Darüber hinaus könnte die Beurteilung der MN der Kinder durch die Eltern verzerrt sein. Eine direkte Befragung älterer Kinder könnte hier Abhilfe schaffen, für jüngere Kinder scheint es derzeit keine praktikablen Alternativen zu geben.

Die Stichproben für die Quer- und Längsschnittanalysen umfassen überwiegend Familien mit mittlerem oder hohem SES. Eine mögliche Ursache könnte in der selektiven Attrition zu finden sein [49]. Dies schränkt die Aussagekraft für Kinder aus Familien mit niedrigem SES ein. In Familien mit niedrigem SES besitzen 75 % der 4‑Jährigen ein eigenes Mobilgerät [14], im Gegensatz zur Allgemeinbevölkerung, in der 14 % der 2‑ bis 5‑jährigen Kinder ein eigenes mobiles Gerät wie ein Tablet besitzen [8].

Die zustande gekommene Stichprobe ist verglichen mit der ursprünglichen Stichprobe verhältnismäßig klein, was wiederum die Aussagekraft der Ergebnisse einschränkt. Zudem sind die meisten Befragten Mütter. Es bestehen Hinweise, dass es keinen Unterschied zwischen Müttern und Vätern in Bezug auf den Zusammenhang zwischen Erziehungsverhalten und externalisierendem Problemverhalten bei Kindern gibt [50]. Jedoch bestehen Unterschiede im Zusammenhang von kindlichen PA mit väterlichem und mütterlichem Stresserleben [51]. In der vorliegenden Studie wurde für das elterliche Geschlecht kontrolliert und es zeigten sich keine signifikanten Unterschiede. Aufgrund der ungleich großen Gruppen der Mütter und Väter sollten die vorliegenden Ergebnisse dennoch nur mit Vorsicht für beide Elternteile interpretiert werden.

Zuletzt muss beachtet werden, dass mithilfe der vorliegenden bevölkerungsbezogenen Daten keine Kausalzusammenhänge untersucht werden konnten. Die Ergebnisse stellen eine Grundlage für weitere longitudinale Analysen dar.

Die Stärke dieser Studie besteht im gleichzeitigen Vorliegen von Quer- und Längsschnittanalysen. Die ursprüngliche Stichprobe ist repräsentativ für Kinder in Deutschland und umfasst ein breites Altersspektrum, das die frühe, mittlere und späte Kindheit abdeckt. Zudem handelt es sich um die aktuellsten vorliegenden Daten für psychische Auffälligkeiten im Kindesalter in Deutschland auf Bevölkerungsebene. Darüber hinaus wurden international etablierte und validierte Instrumente wie der SDQ verwendet. Unserem Wissen nach ist dies die erste Studie, die eine hohe MN, PA, elterliches Verhalten und elterliches Stresserleben in einer Stichprobe und verschiedenen Altersgruppen analysiert. Aus der vorliegenden Studie kann im Zusammenschluss mit Erkenntnissen aus anderen medizinischen, psychologischen und soziologischen Arbeiten ein Gesamtbild zur Betrachtung PA im Kindesalter und möglicher Einflussfaktoren entstehen.

## Fazit und Ausblick

Die Zusammenhänge zwischen einer hohen MN und PA zeigten sich nur im Querschnitt bei Vorschulkindern. Eine hohe MN könnte somit als Indikator für eine nähere Betrachtung der psychischen Gesundheit von Vorschulkindern herangezogen werden. Neben den Wirkmechanismen der Medien, scheinen elterliche Variablen entscheidend für die psychische Gesundheit von Vorschulkindern und Schulkindern zu sein. Insbesondere das elterliche Stresserleben scheint hier eine wichtige Einflussgröße in unterschiedlichen Altersgruppen zu sein, sowohl in quer- als auch in längsschnittlicher Betrachtung. Zukünftige Studien sollten die elterlichen Variablen in Wechselwirkung mit Medienverhalten untersuchen. Zu vermuten ist, dass hohe MN durch eine Reihe von elterlichen Faktoren beeinflusst wird, zu denen neben Stresserleben auch der elterliche Medienkonsum und das Vorliegen von begrenzenden Regeln zur MN gehören könnten. Die gefundenen Zusammenhänge mit elterlichen Variablen zeigten gleiche Relevanz für Jungen und Mädchen unterschiedlichen Alters. Dies spricht dafür, dass, im Sinne von Prävention kindlicher PA, Eltern hinsichtlich ihrer Erziehungskompetenzen gestärkt werden sollten.

## Supplementary Information





## Footnotes

1 Inkonsistentes Erziehungsverhalten ist gekennzeichnet von einem wechselnden Ausmaß an Kontrolle und Reaktionsbereitschaft dem Kind gegenüber [19].

2 Positives Erziehungsverhalten ist charakterisiert durch einen emotional warmen Umgang mit einem hohen Ausmaß an Kontrolle sowie einer kindbezogenen, freundlichen Reaktionsbereitschaft [19].
