# Supplementary material for: Children’s mental health: the role of screen time, parenting behavior, and parenting stress—a secondary data analysis of KiGGS and BELLA data
Source: Bundesgesundheitsblatt Gesundheitsforschung Gesundheitsschutz. 2023 Jun 15;66(7):784–93. [Article in German] doi: 10.1007/s00103-023-03727-y (PMC10328867; doi:10.1007/s00103-023-03727-y)
Supplement: Supplementary file 1 [file 103_2023_3727_MOESM1_ESM.pdf]

Onlinematerial 1: Bivariate Pearson- und Spearman-Korrelationen Vorschulkinder (Querschnitt, n=417)

	PA	MN	SES	G Kind	G Eltern	ES	PE	IE
PA	1	0,16** (0,16**)	-0,12* (-0,11*)	-0,01 (-0,02)	0,08 (0,08)	0,43** (0,34**)	-0,18** (-0,16**)	0,12* (0,12*)
MN		1	-0,18** (-0,19**)	-0,06 (-0,06)	-0,12* (-0,12*)	0,05 (0,03)	-0,06 (-0,06)	0,19** (0,18**)
SES			1	-0,04 (-0,04)	-0,07 (-0,06)	-0,01 (0,01)	-0,01 (-0,04)	-0,10* (-0,10*)
G Kind				1	0,08 (0,08)	0,01 (0,01)	0,06 (0,07)	0,01 (0,01)
G Eltern					1	0,09 (0,07)	0,02 (0,02)	-0,09 (-0,09)
ES						1	-0,27** (-0,25**)	0,42** (0,41**)
PE							1	-0,16** (-0,17**)
IE								1

Legende: *p<0,05; **p<0,01; Spearman-Korrelationen in Klammern, zweiseitig; PA= psychische Auffälligkeiten; MN= Mediennutzung; SES= sozioökonomischer Status; G Kind= Geschlecht des Kindes; G Eltern= Geschlecht des interviewten Elternteils; ES= elterliches Stresserleben; PE= positives Erziehungsverhalten; IE= inkonsistentes Erziehungsverhalten

Onlinematerial 2: Bivariate Pearson- und Spearman-Korrelationen Schulkinder (Längsschnitt, n=239)

	PA T0	PA T1	MN	SES	G Kind	G Eltern	ES	PE	IE
PA T0	1	0,32** (0,32**)	0,10 (0,10)	-0,18** (-0,18**)	-0,14* (-0,14*)	0,04 (0,04)	0,45** (0,39**)	-0,18** (-0,16*)	0,32** (0,28**)
PA T1		1	0,05 (0,05)	-0,04 (-0,04)	-0,12 (-0,12)	0,01 (0,01)	0,37** (0,29**)	-0,08 (-0,07)	0,19** (0,15*)
MN			1	-0,16* (-0,16*)	-0,14* (-0,14*)	-0,05 (-0,05)	-0,04 (-0,07)	-0,05 (-0,05)	0,18** (0,17**)
SES				1	0,04 (0,04)	-0,06 (-0,06)	-0,05 (-0,06)	0,09 (0,08)	-0,01 (-0,08)
G Kind					1	0,06 (0,06)	-0,15* (-0,13*)	0,01 (-0,03)	-0,10 (-0,10)
G Eltern						1	0,01 (0,04)	-0,01 (-0,02)	-0,03 (-0,03)
ES							1	-0,39** (-0,38**)	0,41** (0,39**)
PE								1	-0,18** (-0,16*)
IE									1

Legende: *p<0,05; **p<0,01; Spearman-Korrelationen in Klammern, zweiseitig; PA T0= psychische Auffälligkeiten zur Baseline; PA T1= psychische Auffälligkeiten zum Follow-up; MN= Mediennutzung; SES= sozioökonomischer Status; G Kind= Geschlecht des Kindes; G Eltern= Geschlecht des interviewten Elternteils; ES= elterliches Stresserleben; PE= positives Erziehungsverhalten; IE= inkonsistentes Erziehungsverhalten
